# Intraoperative Airway Management Using Veno-Venous Extracorporeal Membrane Oxygenation for Airway Obstruction From Diffuse Idiopathic Skeletal Hyperostosis at the Thoracic Inlet: A Case Report

**DOI:** 10.7759/cureus.96927

**Published:** 2025-11-15

**Authors:** Yudai Yano, Hirofumi Bekki, Tetsuro Tagawa, Tetsuya Kai, Junichi Fukushi

**Affiliations:** 1 Orthopedics, National Hospital Organization Kyushu Medical Center, Fukuoka, JPN; 2 Orthopedic Surgery, Kyushu Central Hospital of the Mutual Aid Association of Public School Teachers, Fukuoka, JPN; 3 Anesthesiology, National Hospital Organization Kyushu Medical Center, Fukuoka, JPN

**Keywords:** airway obstruction, anesthesiology, diffuse idiopathic skeletal hyperostosis (dish), intraoperative airway control, orthopedics, veno-venous extracorporeal membrane oxygenation (vv-ecmo)

## Abstract

We report a case of airway obstruction caused by diffuse idiopathic skeletal hyperostosis (DISH) at the thoracic inlet, in which surgical treatment was successfully performed using veno-venous extracorporeal membrane oxygenation (VV-ECMO) for intraoperative respiratory support. The patient, a 71-year-old man, presented with progressive dyspnea. CT revealed significant airway narrowing at the thoracic inlet due to bony overgrowth consistent with DISH. Although bone resection was planned, tracheal intubation was considered difficult because of severe airway stenosis. To safely manage ventilation during surgery, VV-ECMO was temporarily initiated for respiratory support. The sternum was then vertically split to relieve the stenosis, allowing successful tracheal intubation and subsequent surgical resection of the osteophytes. This case highlights the potential role of VV-ECMO as a bridge to secure intraoperative airway control in patients with critical airway obstruction caused by space-occupying lesions at the thoracic inlet.

## Introduction

Diffuse idiopathic skeletal hyperostosis (DISH) is a non-inflammatory condition characterized by calcification and ossification of spinal ligaments and peripheral tendon attachments [1]. Common symptoms include neck pain, back pain, and reduced spinal mobility. In rare cases, particularly within otorhinolaryngology, cervical ossification associated with DISH can compress the esophagus and airway, leading to dysphagia and dyspnea [2-6]. Surgical removal of the offending osteophytes is often required to relieve this pressure. However, when airway stenosis is severe, specifically when the tracheal diameter falls below 7 mm, the minimum required for safe intubation, intraoperative airway management becomes a significant challenge.

We report a case of DISH at the thoracic inlet causing critical airway obstruction, in which veno-venous extracorporeal membrane oxygenation (VV-ECMO) was employed to maintain adequate oxygenation during airway securing and osteophyte resection. This case contributes to the growing body of literature on airway management strategies in high-risk surgical scenarios.

## Case presentation

The patient was a 75-year-old man with no significant medical or family history. He had experienced progressive difficulty breathing for two years prior to his initial consultation. Physical examination revealed no abnormal heart or lung sounds.

**Figure 1:**
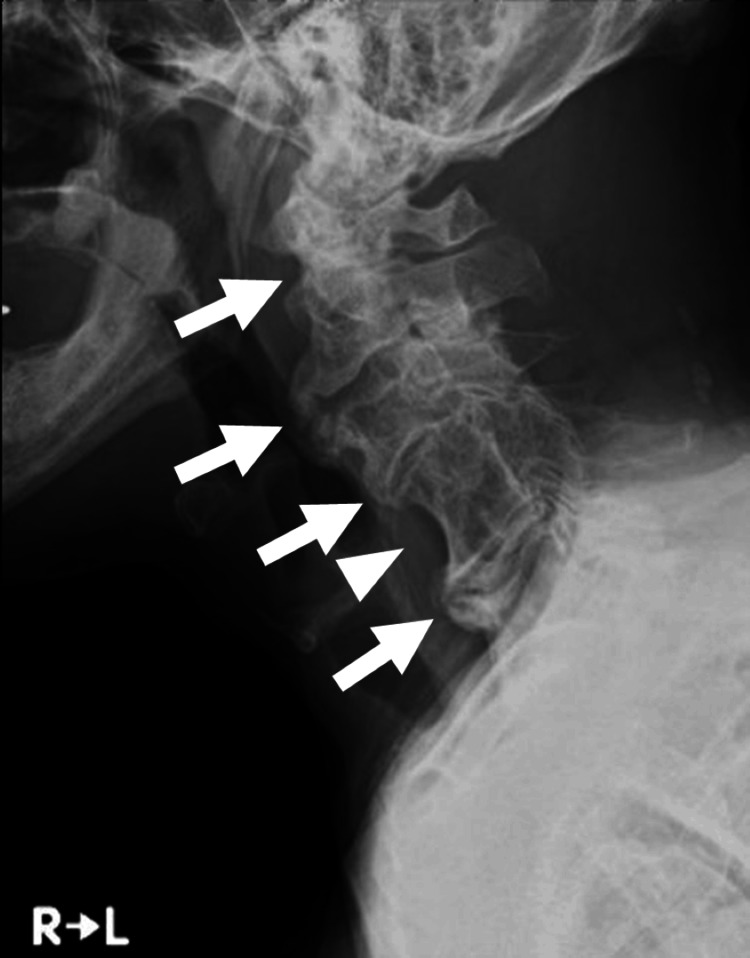
Plain X-ray of the cervical spine Degenerative changes are seen across multiple cervical vertebrae (arrow). Vertebral body fusion is observed at the C4-C5 level (arrowhead).

**Figure 2:**
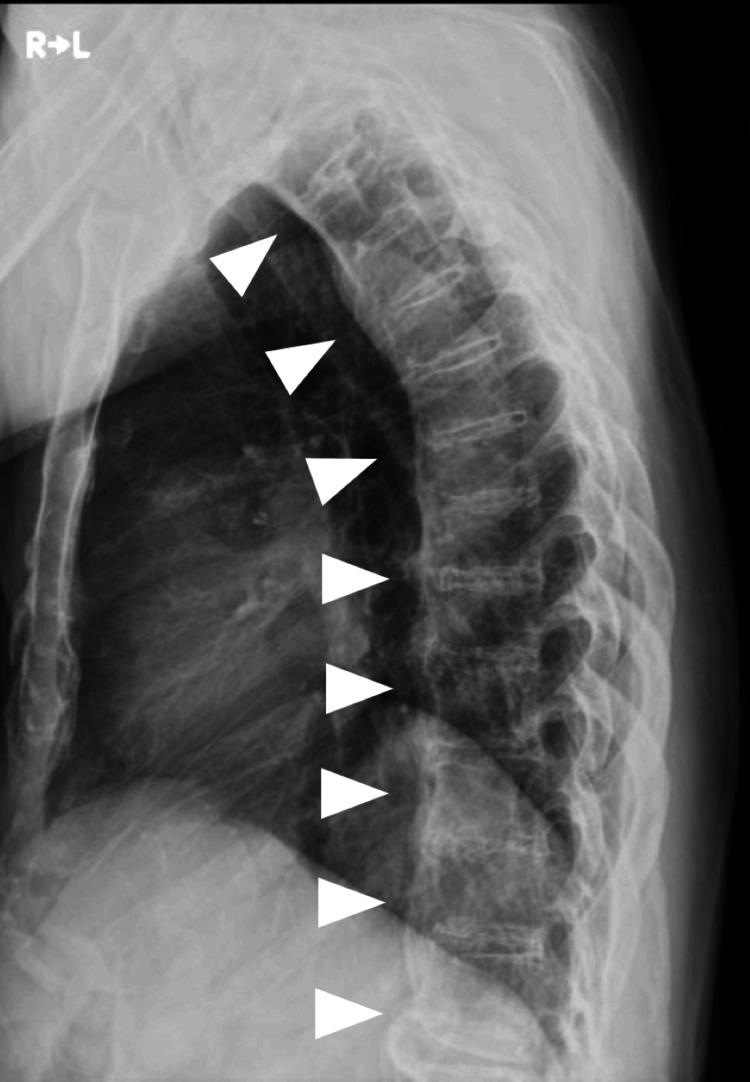
Plain X-ray of the thoracic spine Fusion of adjacent vertebral bodies is observed from the thoracic to lumbar spine due to ossification of the anterior longitudinal ligament (arrowhead).

Pulmonary function testing demonstrated a mixed ventilatory disorder, with a vital capacity (%VC) of 68.3% and a forced expiratory volume in one second (FEV₁%) of 40.3% (Table 1). Plain radiography showed degenerative changes across multiple cervical vertebrae, with fusion observed at the C4-C5 level (Figure 1). In the thoracic and lumbar spine, fusion of adjacent vertebral bodies due to ossification of the anterior longitudinal ligament was noted, consistent with DISH (Figure 2).

**Table 1:** Pulmonary function test results Pulmonary function testing demonstrated a mixed ventilatory disorder. %VC [%] = Actual vital capacity [mL] / Predicted vital capacity [mL] * 100; FEV₁% [%] = forced expiratory volume in one second [mL] / forced vital capacity [mL] * 100

Parameter	Value (patient)	Reference value
%VC [%]	68.3	≥80: normal; <80: restrictive ventilatory disorder
FEV₁% [%]	40.3	≥70: normal; <70: obstructive ventilatory disorder

**Figure 3:**
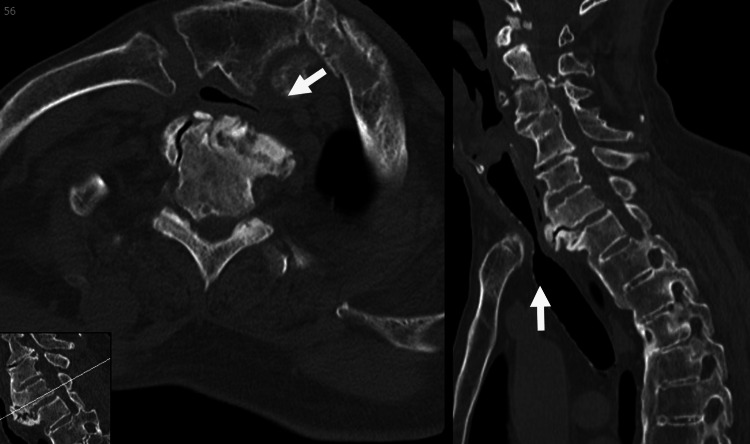
Plain CT of the upper thoracic level The arrow indicates the space-occupying lesion and tracheal stenosis at the thoracic inlet.

CT revealed prominent anterior ossification at the cervicothoracic junction, accompanied by bony proliferation on the posterior surface of the sternum. These structural changes resulted in anteroposterior compression of the trachea (Figure 3). Based on these radiological findings, the patient was diagnosed with airway stenosis due to DISH and sternal bone overgrowth. Surgical resection of the bony structures was subsequently planned.

**Figure 4:**
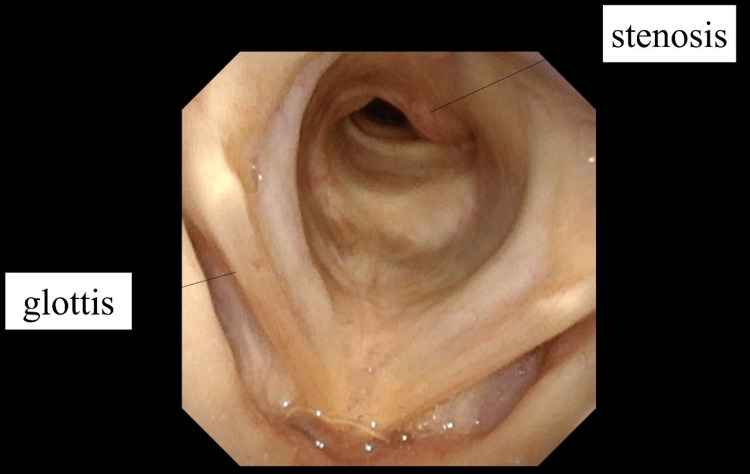
Bronchoscopy Extensive subglottic airway stenosis is observed below the glottis.

Preoperative bronchoscopy revealed extensive subglottic airway stenosis (Figure 4). A 2.8 mm diameter bronchoscope was able to pass through the narrowed segment, and the anteroposterior diameter of the airway was estimated at approximately 5 mm. Given the severity of the stenosis, both orotracheal intubation and tracheostomy were deemed unfeasible, the latter due to the location of the obstruction around the Th1-2 vertebral levels.

To enable safe intraoperative airway management and minimize bleeding risk, VV-ECMO was selected, providing short-term respiratory support without the need for systemic anticoagulation. VV-ECMO was initiated while the patient was awake. General anesthesia was subsequently induced using fentanyl, remifentanil, and propofol. A laryngeal mask airway was placed to facilitate ventilation and maintain oxygenation. Thoracic surgeons then performed a vertical sternotomy to relieve anterior tracheal compression. Following decompression, standard endotracheal intubation was successfully performed, and VV-ECMO support was discontinued. The time from ECMO initiation to intubation was 20 minutes, and ECMO was weaned 30 minutes after intubation.

Osteophytes compressing the trachea were removed to the greatest extent possible. No heparin was administered intraoperatively, and blood loss was minimal, totaling 160 mL. The surgical procedure lasted one hour and 13 minutes.

**Figure 5:**
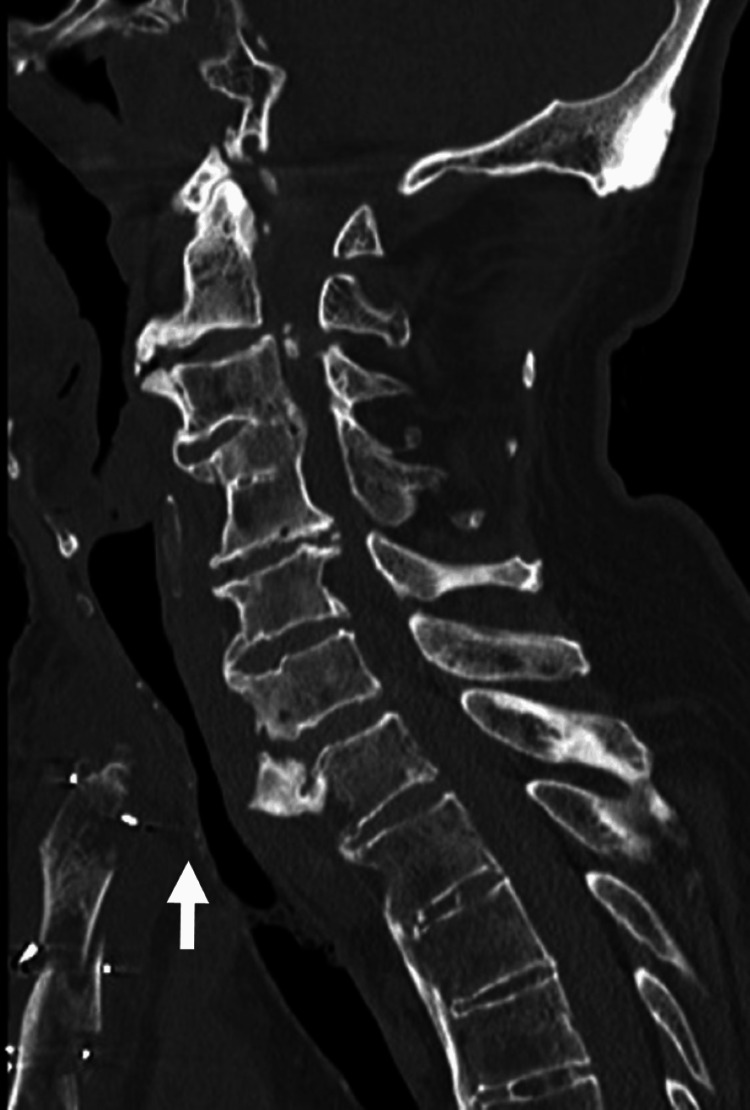
Postoperative CT of the upper thoracic level The arrow indicates that most of the bony overgrowth has been resected, with relief of tracheal stenosis.

Due to the risk of postoperative tracheal edema, the patient was admitted to the intensive care unit with the endotracheal tube in place. He was extubated on postoperative day 2. The patient’s dyspnea improved significantly, and follow-up pulmonary function testing showed an increase in FEV₁% from 40.3% to 66.9% (Table 2). Postoperative CT imaging confirmed that most of the bony overgrowth had been successfully resected (Figure 5).

**Table 2:** Pre- and postoperative pulmonary function tests Postoperative pulmonary function testing demonstrated improvement in FEV₁%. %VC [%] = Actual vital capacity [mL] / Predicted vital capacity [mL] * 100; FEV₁% [%] = forced expiratory volume in one second [mL] / forced vital capacity [mL] * 100

Parameter	Before surgery	After surgery	Reference value
%VC [%]	68.3	65.4	≥80: normal; <80: restrictive ventilatory disorder
FEV₁% [%]	40.3	66.9	≥70: normal; <70: obstructive ventilatory disorder

A postoperative wound infection occurred but was successfully managed with surgical debridement and antibiotic therapy.

## Discussion

DISH is a non-inflammatory condition characterized by calcification and ossification of spinal ligaments as well as peripheral tendon or ligament attachment sites. The most widely accepted diagnostic criteria were established by Resnick et al. in 1976: (1) “flowing” calcification and ossification along the anterolateral aspects of at least four contiguous vertebral bodies; (2) relative preservation of intervertebral disk height in the involved areas without significant radiographic evidence of degenerative disk disease; and (3) absence of apophyseal (facet) joint ankylosis and sacroiliac joint erosion, sclerosis, or fusion [1].

Symptoms often include neck or back pain and reduced spinal mobility. In rare cases, ossified lesions may mechanically displace the esophagus or trachea, resulting in dyspnea or dysphagia [2-6]. To the best of our knowledge, no prior reports have described airway management in symptomatic ossified lesions specifically located at the thoracic inlet.

At most thoracic levels, surrounding soft tissues, such as the lungs, provide a degree of spatial flexibility, allowing the trachea or esophagus to shift in response to compression. In this case, however, simultaneous ossification of both the anterior vertebral bodies and the posterior surface of the sternum created a fixed, confined space that prevented such displacement. This anatomical constraint resulted in critical tracheal compression and progressive dyspnea.

Airway management posed a significant challenge. Preoperative bronchoscopy demonstrated severe airway narrowing, permitting passage of only a 2.8 mm diameter fiberoptic bronchoscope. Conventional endotracheal intubation was therefore considered unsafe. While the Japan Advanced Trauma Evaluation and Care guidelines recommend emergency surgical airway procedures such as cricothyroidotomy or cricothyroid puncture in difficult airway cases, these interventions were inappropriate here because they would secure the airway proximal to the site of obstruction at the thoracic inlet.

Given these constraints, we elected to initiate ECMO to provide temporary respiratory support, allowing safe induction of anesthesia and decompression sternotomy prior to securing the airway.

ECMO is a system that supports gas exchange by removing carbon dioxide and oxygenating blood outside the body, returning it to circulation through venous or arterial access [7]. In this case, VV-ECMO was used, which supports oxygenation but does not provide circulatory support. This system typically involves draining blood via the femoral vein and returning it through the internal jugular vein. VV-ECMO provides antegrade blood flow with fewer complications than veno-arterial ECMO. Clinicians must, however, be mindful of the risk of intracircuit recirculation, in which oxygenated blood is withdrawn again before reaching systemic circulation, thereby reducing ECMO efficiency [7].

Since our objective was to maintain oxygenation only for the short duration needed to relieve airway compression, VV-ECMO was chosen. Oxygenation was successfully maintained throughout the procedure, and endotracheal intubation was achieved following decompression. Yanada and Toda previously reported the effectiveness of VV-ECMO during surgical management of bilateral pneumothorax, further supporting its utility in short-term intraoperative respiratory support [8].

The Extracorporeal Life Support Organization guidelines recommend systemic anticoagulation, typically with heparin, during ECMO to prevent circuit thrombosis [7]. In this case, however, we prioritized hemostasis, surgical field visibility, and prevention of postoperative hematoma. Therefore, ECMO was performed without anticoagulation for approximately 15 minutes until the airway was secured. Although the omission of anticoagulation during ECMO remains debated, no definitive consensus exists regarding safe duration or thresholds. Robba et al. concluded that initial ECMO management without anticoagulation may be a viable option in patients at high risk of bleeding [9].

Our experience suggests that VV-ECMO can serve as a valuable adjunct for intraoperative airway management in cases of critical tracheal obstruction caused by compressive lesions at the thoracic inlet, such as those due to DISH or tumors.

## Conclusions

We report a case of DISH at the thoracic inlet causing critical airway obstruction, in which intraoperative airway management was successfully achieved using VV-ECMO. VV-ECMO provided essential respiratory support during the brief but critical period before tracheal intubation, enabling safe surgical decompression. This approach may be a valuable option in similar cases where conventional airway management is not feasible due to severe anatomical obstruction.
